# Induced Mutations in Yeast Cell Populations Adapting to an Unforeseen Challenge

**DOI:** 10.1371/journal.pone.0111133

**Published:** 2014-10-23

**Authors:** Lindsay S. Moore, Wu Wei, Elad Stolovicki, Tamar Benbenishty, Stefan Wilkening, Lars M. Steinmetz, Erez Braun, Lior David

**Affiliations:** 1 Department of Physics & Network Biology Research Laboratories, Technion-Israel Institute of Technology, Haifa, Israel; 2 Stanford Genome Technology Center, Palo Alto, California, United States of America; 3 Department of Animal Sciences, The Hebrew University of Jerusalem, Rehovot, Israel; 4 European Molecular Biology Laboratory, Genome Biology Unit, Heidelberg, Germany; University of Strasbourg, France

## Abstract

The modern evolutionary synthesis assumes that mutations occur at random, independently of the environment in which they confer an advantage. However, there are indications that cells facing challenging conditions can adapt rapidly, utilizing processes beyond selection of pre-existing genetic variation. Here, we show that a strong regulatory challenge can induce mutations in many independent yeast cells, in the absence of general mutagenesis. Whole genome sequencing of cell lineages reveals a repertoire of independent mutations within a single lineage that arose only after the cells were exposed to the challenging environment, while other cells in the same lineage adapted without any mutation in their genomes. Thus, our experiments uncovered multiple alternative routes for heritable adaptation that were all induced in the same lineage during a short time period. Our results demonstrate the existence of adaptation mechanisms beyond random mutation, suggesting a tight connection between physiological and genetic processes.

## Introduction

Changes in the environment impose challenges that, unless resolved by the organism, might drive its population to extinction. The current consensus based on the modern synthesis separates physiological processes from evolutionary adaptation. The former are transient responses in an individual, while the latter relies on selection of genetic variation that accumulates in the population independently of the selective environment [1]. Recent research however, has highlighted physiological and epigenetic processes beyond genetics that could respond directly to environmental cues and facilitate inheritance of adaptive traits [2]–[4]. Currently, however, there is no framework that connects genetics to other processes that might promote the occurrence of specific mutations without an increase in the overall mutation rate.

Notwithstanding the success of the Neo-Darwinian framework of adaptation based on random mutation and selection, this framework alone cannot explain the entire spectrum of processes that can lead to inherited adaptation. In particular, there are indications that the rate of beneficial mutation is low [5]–[7] and thus might be a problem for survival in unstable environments. Alternatively, there are some indications that mutagenesis can be induced under stressful conditions [8], [9]. However, since deleterious mutations are more likely than beneficial ones, there are limits to how pervasive this solution can be, and most efforts in this area have explored the constraints on increased mutation rates in response to a challenging environment [10]–[12]. In this paper, we show by direct comparison of the genomic sequences of adapting cells within a single lineage that mutations are induced by the challenging environment within a strictly limited time window. Furthermore, we demonstrate that mutations can emerge in specific genes at a very high rate, but not due to general mutagenesis in these lineages.

We have previously developed an experimental system to study adaptation of genome-rewired yeast cells to an unforeseen challenge. *HIS3*, an essential gene in the histidine biosynthesis pathway has been placed under the exclusive regulation of the *GAL* system, responsible for galactose utilization [13]. These genome-rewired cells are faced with multiple challenges, primarily those of gene regulation, and most notably the repression of *HIS3* in glucose based medium. We have previously shown that such populations adapt quickly, within ∼10 generations, to grow exponentially in glucose medium lacking histidine (Glu-his) and that this adaptation is inherited for many generations at the population level [13], [14]. Moreover, detailed experiments have shown that, on average, 50% of the naïve cells adapt on Glu-his plates, suggesting that the rapid adaptation is not due to selection of a rare pre-existing subpopulation but rather due to the availability of multiple adaptation solutions [14]. In some adapted populations, mutations in regulatory elements of the *GAL* system, such as the repressor *GAL80*, were found, but by themselves these mutations were not sufficient to stabilize the adapted phenotype [15]. Naïve, rewired cells with *GAL80* mutation allele replacement do not grow exponentially in Glu-his medium although as a population they are more successful in this environment than naïve rewired cells with intact *GAL80*. These findings set the stage to address two important questions: First, given that the challenging environment induced the phenotypic adaptation process, did the environment also induce the mutations that arise in some of the cells? Second, does the observed, remarkable, high rate of adaptation in our experiments also imply an exceptionally high rate of mutation? To address these questions, we analyzed the mutation repertoire in lineages originating from isolated single cells following their adaptation to the glucose medium.

## Results

**Figure 1:**
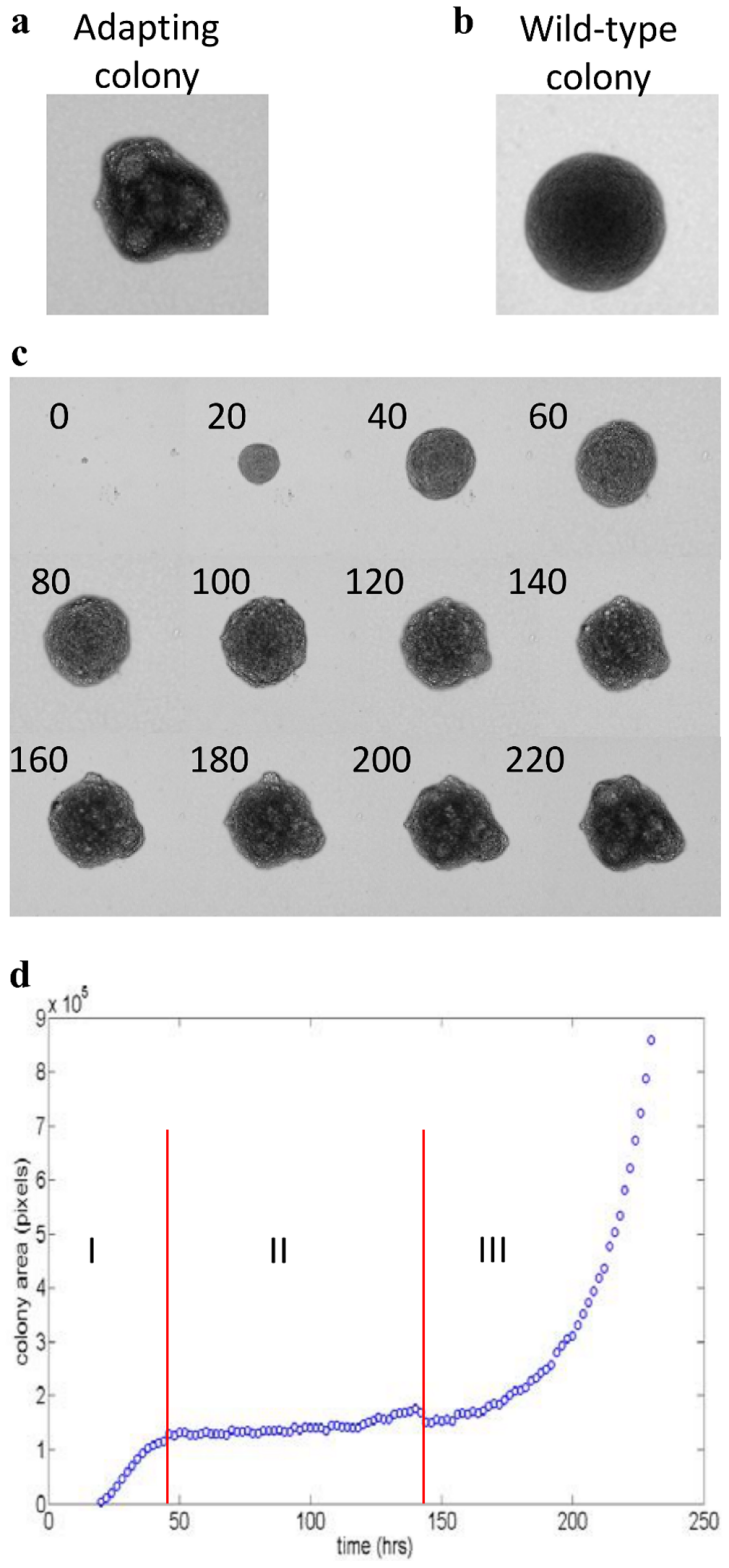
Time-lapse microscopy of lineages that started from a single rewired cell. Single, naïve, rewired cells plated on Glu-his agar medium were imaged by bright-field microscopy. (a) Colony morphology of an adapting lineage that started from a single rewired cell. (b) Colony morphology of a lineage that started from a single wild-type cell. Exponentially growing, wild-type colonies have uniform, circular colony morphology. (c) The development over time (in hours) of an adapting colony on a Glu-his plate. Typical microscopy of adapting, rewired colonies shows foci of growth starting around 100 hours post-plating. (d) Change in approximate colony area (in pixels) over time (in hours) exhibits three phases of colony growth: phase-I with exponential growth, phase-II with almost no increase in colony area, and phase-III with resumed colony growth. Images were obtained at two hours intervals from a second adapted colony.

We first measured the course of adaptation in lineages originating from single cells by following the growth of individual, adapting colonies using time-lapse microscopy. A naïve, rewired cell that had never before been exposed to Glu-his was placed on a Glu-his agar plate after growth in galactose medium lacking histidine (Gal-his). Previous measurements have shown that ∼50% of naïve cells plated this way will grow an adapted colony within 20 days of plating. The growth morphology of one of these adapting colonies is non-uniform during adaption, when compared to an exponentially growing, wild type colony (Fig. 1a vs. 1b). Of particular interest are the multiple growth-centers distributed throughout the colony that emerge after several days (Fig. 1c). To measure the time-course of the colony growth, images were taken every one or two hours throughout adaptation (For an example of a colony see Movie S1 and Fig. 1c), and the area of the colony was estimated using standard image analysis techniques. Figure 1d shows the size of a typical colony as a function of time. The adaptation dynamics of cells exposed to Glu-his is characterized by distinct phases: an exponential increase in cell number immediately after plating creating a micro-colony (phase I), followed by a long period of almost no cell divisions (phase II). Remarkably, after 2–3 days of arrest, growth resumes slowly, with staggered foci of cell division distributed throughout the micro-colony (Movie S1, 120–220 hrs). These foci are initiation cores of adapted sub-lineages of the original mother cell, but do not immediately exhibit fast exponential growth (phase III). The phases of adaptation of single lineages are similar to those observed at the population level in batch and chemostat cultures [13], [14], [16], and can be seen in repeated microscopy measurements of different lineages. The growth dynamics (Movie S1) reveal that adaptation can occur in more than one cell within each lineage after a significant time of cell-division arrest. The adaptive growth that starts within the micro-colony and the large number of cells per plate that eventually adapt preclude the possibility that adaptation was achieved by selection of a rare pre-existing variant; a conclusion consistent with the dynamics observed at the population level, and with the large fraction of adapting cells [13], [14].

**Figure 2:**
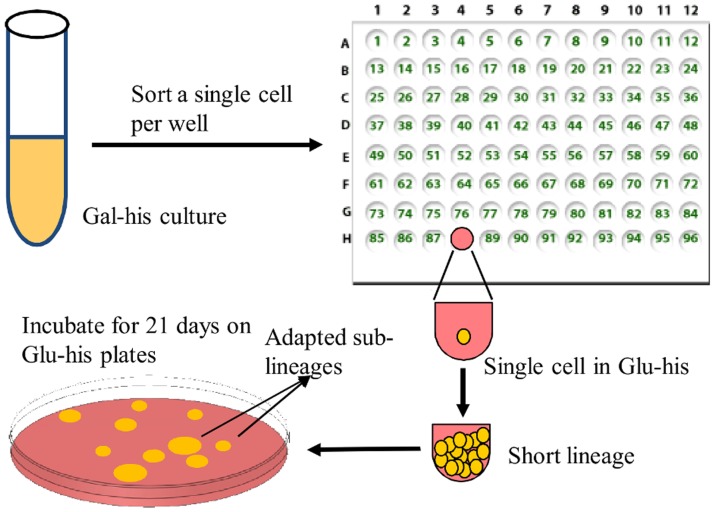
Schematic of lineage separation technique. Single cells were sorted from a growing Gal-his culture of rewired cells into each well of a 96-well plate containing 200 µL Glu-his medium. The 96-well plate was incubated for 48–72 hours at 30°C, shaking at 350 rpm until a halt in cell division. All cells from a single well were then spread on a Glu-his agar plate and incubated for 21 days at 30°C. Each plate contained only a single lineage, and each colony that grew represented an independently-adapted sub-lineage.

To further explore the variety of adapting sub-lineages originating from a single mother cell we devised a measurement setup that would allow us to isolate individually adapting cells during phase-II. From a growing culture of rewired cells in Gal-his, a single cell was sorted into each well of a 96-well plate containing Glu-his medium (Fig. 2). The 96-well plate was incubated for 48–72 hours until the lineage in each well had completely reached the end of phase-I, as marked by the effective halt in cell division. At this stage, all cells from a single well were spread on a Glu-his agar plate, physically isolating all daughter cells of the lineage (lineages at the end of phase-I contained 400–3000 cells). Plates were then incubated for 21 days. Notably, no mature colonies were observed on the plates in the first five days of incubation, indicating that after completing phase-I in the wells, cells were not yet adapted (otherwise colony formation would have immediately followed single-cell plating). Given more incubation time, a subset of the plated sub-lineages resumed growth similar to the staggering division foci observed in the time-lapse microscopy and formed visible, adapted colonies. Fifty-six lineages, each originating from separate wells, were grown in this way from a total of three different batch cultures sorted into 96-well plates. The number of adapted colonies per lineage was variable, with an average of 19 independently-adapting sub-lineages per lineage (Figure S1).

In contrast to adaptation of populations in batch or chemostat cultures, adaptation on agar plates lacks competition among sub-lineages, and thus provides a faithful representation of the spectrum of adaptation solutions. We have previously identified the gene *GAL80* as a common locus for mutations in long-term adaptation experiments, suggesting that the mutation either arises early in adaptation or that it confers some selection advantage [15]. Having eliminated the possible selective advantage of mutations in this gene by the plate assay, we measured the frequency of *GAL80* mutations in 11 independent lineages by sequencing this locus in all of the 192 adapted colonies belonging to these lineages. Eight of the 192 adapted colonies (∼4.2%) were found to have a mutation in *GAL80* and all these different mutations were non-synonymous (Table 1).

**Table 1:** *GAL80* mutations identified in lineages of adapted clones.

	Lineage	Number ofadapted clones	Mutated clone(sub-clone)	DNApolymorphism^1^	Proteinpolymorphism^2^	Remarks
1	1	24	6	1174, C >T	392, Q>*	Heterogeneous colony bySanger sequencing
2	1	24	8(14)	457, C>G	153, R>G	Co-existed in onecolony with 3
3	1	24	8(12)	281, 1bp deletion	Frame shift and *	Co-existed in onecolony with 2
4	1	24	10	421, G>T	141, E>*	Not detected bySanger sequencing
5	1	24	18	1189, G >T	397, E>*	
6	2	19	5	457, C>G	153, R>G	
7	3	16	9	458, G>C	153, R>P	
8	4	32	8	893, G>A	298, G>D	

1 The position in base pairs relative to the first base of the start codon, the original and new nucleotides.

2 The protein sequence position, the original and new amino acids. * denotes a change into stop codon.

In general, the incidence of mutations in *GAL80* is much higher than expected from previously estimated rates of random mutations in yeast which were on the order of 10^−8^ −10^−10^ per base per generation [5], [6], [17], indicating that these mutations emerged due to an unusual process. Indeed, we found colonies with and without a *GAL80* mutation within the same lineage and different mutations were found between and even within lineages. Thus, these mutations occurred independently, in different colonies only after the daughter cells of each lineage were separated on the Glu-his plates. Seven of the mutations were found by Sanger sequencing. Since reliable base calling in this method requires most sequenced molecules to have an identical base at any given position, the identification of *GAL80* mutations indicates that these mutations emerged within the first 1–3 generations of adapted growth in the colony. If the mutation emerged in the first adapted cell, all cells in the resulting colony, being daughters of this first cell, will carry this mutation. If the mutation emerged in one of the four cells resulting from the first two divisions of the first adapted cell, ¼ of the cells in the mature colony will carry this mutation. Mutations emerging later than that would be difficult to detect by Sanger sequencing.

More importantly, emergence of the mutations close to the time of initial adapted growth rules out the option that the high incidence of *GAL80* mutation was a result of selection of a few rare, advantageous variants that existed in the cell population prior to spreading on Glu-his plates. With respect to the role of selection in this adaptation process, the mutation emergence time is consistent with the long time required for adapted colonies to grow on the Glu-his plates, both indicating against selection of pre-existing variants. The negligible role of selection is even clearer when considering that remarkably, one adapted colony contained adapted cells with and without a *GAL80* mutation, while another colony contained cells with one *GAL80* mutation along with cells containing a second mutation (Table 1). This gives further support to the fact that the different mutations emerged independently, very early upon the resumption of growth of the sub-lineage that formed this colony and certainly after plating.

Our results from adaptation in lineages therefore suggest that either these mutation were generated as part of a general mutagenesis process, or that they were induced due to a directed physiological process after exposure to the challenging environment. To evaluate the possibility of general mutagenesis and to verify the independence of the adapting sub-lineages, we performed whole genome sequencing of two lineages. Lineage 1 included 17 independently adapting colonies, two with a mutation in *GAL80*, and 15 without, while lineage 2 included nine independently adapting colonies, one with a mutation in *GAL80*, and eight without. Two additional clones with *GAL80* mutations from other lineages were also sequenced. The analysis of the sequencing results was limited to adapted strains that had genome coverage greater than five reads per base in 80% or more of their nuclear genome. Mitochondrial DNA mutations were excluded from the analysis. To identify mutations, the sequences of the adapted strains were compared with the sequence of the naïve ancestral strain YPH499 plus the plasmid.

**Figure 3:**
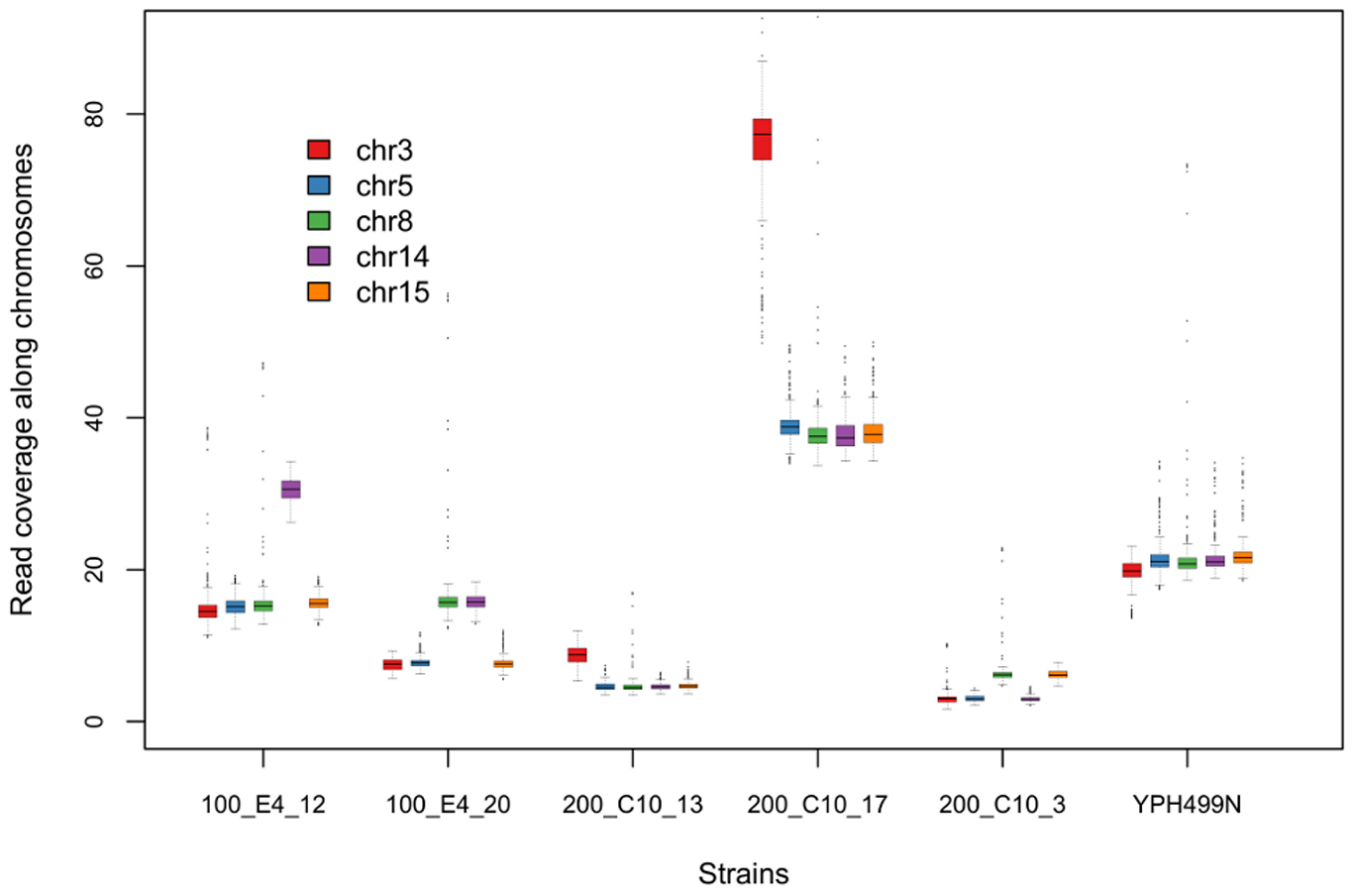
Chromosome coverage for strains with duplications. Determination of chromosome duplication was based on comparing the average coverage per chromosome to the averages of the rest within each strain. Chromosomes III, VIII, and XIV were each duplicated in two strains while chromosome XV in only one. The ancestral YPH499N control is shown without any duplication. Boxplots of read coverage in 500 random windows with a size of 10,000 bp each were plotted. In each window, mean value of the read coverage of each position inside the window was calculated.

**Figure 4:**
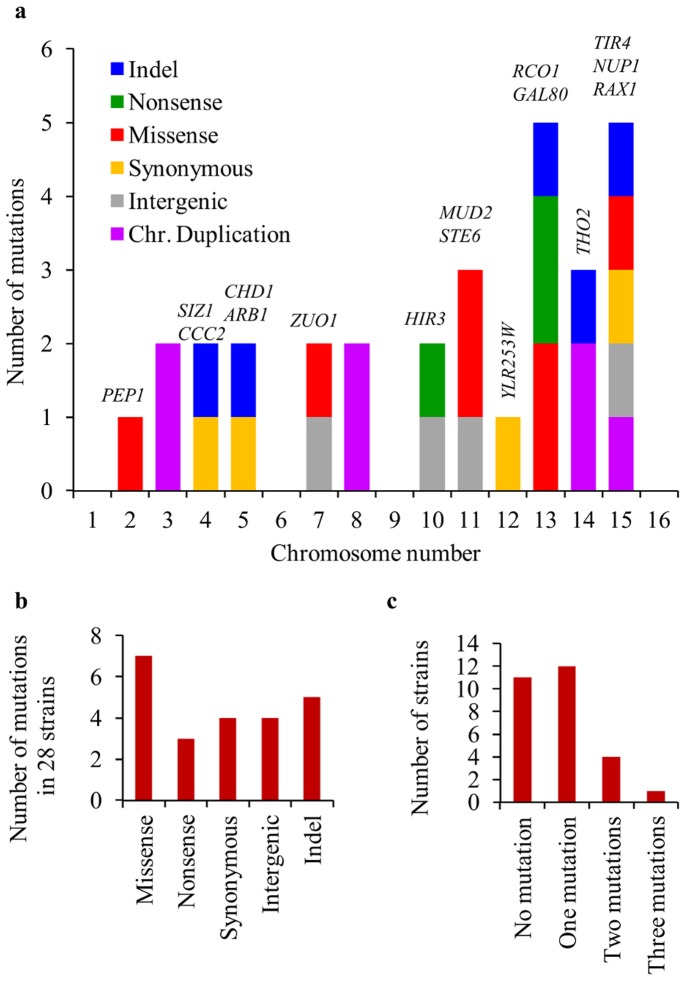
Distribution of mutation types and locations. Mutations in nuclear DNA from the 28 adapted strains from two lineages contained indels, SNPs and chromosome duplications. A) Distribution by chromosome of each mutation type. B) Distribution of the different kinds of SNPs and indels in the 28 strains show no enrichment of a single mutation type. C) Distribution of the number of mutations per strain (excluding chromosome duplication) across the 28 adapted strains. Most genomes contained one mutation or no mutations at all.

Comparing the 28 adapted strains to the naïve reference strain, three types of polymorphisms were found: chromosome duplications, insertions/deletions (indels) and single nucleotide polymorphisms (SNPs). First, we found that five strains had one or two duplicated chromosomes (Fig. 3). Chromosomes III, VIII, and XIV were each duplicated in two strains while chromosome XV in only one. Second, after removing uncertain indels in low complexity and repetitive sequences, five indels were found inside genes (Fig. 4a and Table S1). Three of the indels kept the gene sequence in-frame while two interrupted the open reading frame (ORF) causing a frame shift and premature stop codons. Finally, we found 18 SNPs in the 28 sequenced genomes: four intergenic, four synonymous, three nonsense and seven missense (Fig. 4b and Table S1). Excluding chromosome duplications, one genome contained three mutations, four contained two, twelve contained one mutation and, remarkably, eleven strains had no mutations at all (Fig. 4c). The distribution of mutations in these sub-lineages, and lack of repetition corroborates the conclusions from the *GAL80* sequencing that the mutations occurred independently post plating.

## Discussion

By separating sub-lineages on plates after completion of the initial growth, we clearly demonstrated that not only was the adapted phenotype gained after this first growth phase in the challenging condition, but also the genetic changes were induced after introduction of the challenge. Thus, the adaptation of these rewired cells did not rely on selection of a few, rare genetic variants that pre-existed prior to the challenge imposed by the environment. Given the large proportion of independently adapting cells, we analyzed the repertoire of mutations in order to detect signs of general mutagenesis that might underlie this prevalent adaptation. Excluding chromosome duplications, the number of mutations per genome was remarkably low (on average only 0.82 mutations per genome). Therefore, there was no evidence for a general mutagenesis process that could explain the high incidence of independent *GAL80* mutations. The per-base mutation incidence can be estimated by dividing the sum of all SNPs and indels (23 mutations in total) by the sum of bases in 28 nuclear genomes. All together, the per-base probability for a mutation was 6.8×10^−8^ and the incidence of each polymorphism type was similar (Table 2). These results are similar to those found in previous studies [5], [6], [17]. Thus, neither the number of mutations per genome, nor the normal per-base probability for mutations indicates the existence of increased mutagenesis. Furthermore, the strains with the known mutation in *GAL80* did not have the large number of additional mutations that would be required to explain the high incidence of mutation in that gene under a scenario of general mutagenesis.

**Table 2:** The per-base mutation incidence in adapted strains by mutation types.

Mutation type	Per-base incidence
Intergenic	1.18E-08
Synonymous	1.18E-08
Missense	2.07E-08
Nonsense	8.88E-09
Indel	1.48E-08
All types	6.8E-08

This system of genome-rewired cells exposed to an unforeseen challenge has uncovered a process of phenotypic adaptation induced at unprecedented rates of 50%, on average [13], [14]. Analysis and comparison of adapting sub-lineages descending from a single mother cell showed that specific mutations were induced in a narrow time window after exposure to the challenging environment, but were not a result of general mutagenesis. The existence of directed mutations and the current understanding of stress-induced mutations are subjects of fierce debates [8], [9], [18], [19]. *GAL80* comprises approximately 0.0001 of the genome sequence and the incidence of mutations in that gene was 0.042. Thus, our results indicate that during the time at which cells experienced the regulatory stress of the Glu-his environment, some genomic regions had a higher chance for mutations. This suggests that the mutations were directed by physiological processes rather than occurring at random in the genome. The puzzling occurrence of directed mutations in our experiments suggests a tight connection between the physiological processes that were induced by the environment and the genetics of the adapted cells.

Additionally, our previous work [15] showed that those mutations are neither necessary nor sufficient for the stabilization of the adapted phenotype. Specifically, while genetically clonal, adapted cells maintain growth in Glu-his medium for many generations as a population, individual adapted cells within this population, including those with a *GAL80* mutation, may lose this growth ability [15], [20]. These results suggest that the process of adaptation was accompanied by heritable epigenetic changes of yet unknown nature. This observation was supported by our finding here that eleven of the sequenced sub-lineages did not contain any genetic mutations. Since sub-lineages with heritable, non-genetic adaptation processes occur in the same lineages as adapting cells with a directed mutation, it is clear that the physiological, cellular response to the environmental challenge results in a variety of regulatory and genetic changes. Given the breadth of successful adapted cell states, it is surprising that there are genomic changes that seem to be specifically targeted to a single gene. While we do not yet know the mechanism for this targeting, it is possible that different epigenetic states at different genomic positions will render them more prone to mutations. Our findings call for further work to elucidate the dynamic crosstalk between physiological processes, including epigenetics, and mutations as a measure of coordination between environment and phenotype in the context of natural evolution and disease states.

## Materials and Methods

### Yeast Strain

Experiments were carried out using a strain of *S. cerevisiae* with *HIS3* under exclusive regulation of p*GAL1*. Haploid yeast strain YPH499 [*MATa, ura3–52, lys2–801, ade2–101, trp1-Δ63, his3Δ200, leu2Δ1*] carrying the plasmid pESC-LEU (Stratagene) containing the p*GAL1*-p*GAL10* divergent promoter with *HIS3* under p*GAL1* as described in [13]. Cloning was done by standard methods and was confirmed by fragments analysis and/or by direct sequencing. Transformation was done with the lithium acetate method.

### Media

The growth medium for all batch cultures was either standard minimal medium made of 1.7 g/liter yeast nitrogen base without amino acids and ammonium sulfate, 5 g/liter ammonium sulfate, 1.4 g/liter amino acid dropout powder (without tryptophan, histidine, leucine, and uracil; Sigma, St. Louis), 0.006 g/liter L-tryptophan, and 0.003 g/liter uracil plus 2% of either glucose or galactose; or standard YPD rich medium containing 1% yeast extract, 2% peptone, 2% glucose. Agar plates are made with the same growth medium plus 2% agar.

### Preparation

At the beginning of each microscopy and 96-well plate experiment, naïve, rewired cells from a frozen stock were spread on galactose agar plates lacking histidine and leucine. After several days of incubation at 30°C, a single colony was dispersed in 10 mL of Gal-his medium and incubated at 30°C shaking at 190 rpm. The batch culture was diluted 1∶100 as needed to maintain an OD600<1.0 for three days before starting the experiment.

### Microscopy

Samples were diluted to an OD of 1e-4 and spread on agar plates made with glucose minimal medium lacking histidine and leucine. Plates were incubated for 10–20hours at 30°C to allow a few cell divisions to occur. An agar plate was placed on a 4″ plate holder on a Zeiss inverted microscope fitted with an OXO incubator at 30°C and a homemade humidifier. Individual colonies were identified by eye and imaged using Zeiss Axiovision software together with an ASI stage that allows for acquisition of many positions per time-point. Colonies were imaged every one or two hours and the focus was corrected manually every eight hours for the duration of the imaging. Images were analyzed using a homemade segmentation routine (Matlab) to estimate colony area.

### 96-well plate assay

Two-hundred micro-liters of Glu-his medium were put in each well of a 96-well plate. Naïve yeast cells were deposited, 1 cell per well, using FACS. The 96-well plates were closed with the lids that came with the plates to allow air flow into the samples. The plates were incubated at 30°C for 48–72 hours under a bell jar to maintain humidity, until the lineage in the well had ceased exponential growth. Wells were verified to contain populations using light microscopy before all 200 micro-liters was pipetted onto the surface of a 9 cm Glu-his agar plate and spread with sterile glass beads.

### 96-well plate assay controls

We verified that one cell was being deposited per well of the plate by depositing single cells by FACS into wells of a 96-well plate that had been previously filled with YPD agar. The 96 well plates were incubated for 12 hours and then scanned using light microscopy to determine how many micro-colonies were growing in each well. Two 96-well plates were filled with YPD agar and a single cell was plated in each well. After 12 hours incubation, 1 well had foreign contamination, 2 wells had bubbles interrupting the surface of the agar, making imaging impossible, and 1 well had 2 micro-colonies. Therefore, the error rate of single-cell plating is 1/189, or 0.05%.

The average number of cells in each well during phase-II was determined by plating the entire contents of 16 phase-II wells onto 16 YPD agar plates. The plates were incubated at 30°C for 3 days and then counted. The mean number of colonies that grew was 1357, with a standard deviation of 676 and an estimated standard error of 169.

Two single-lineage Glu-his agar plates were inspected with brightfield microscopy after 21-day incubation using a 10x objective to count the number of colonies that were invisible to the naked eye. The first plate had approximately 700 cells in 27 small colonies of 3–100 cells in addition to 8 colonies that were adapted and visible. The second plate had approximately 300 cells in 7 small colonies of 3–150 cells in addition to 7 adapted colonies.

### Sanger Sequencing

All adapted colonies of each lineage were picked from the agar Glu-his plates and stored in 96-well plates containing YPD rich medium and glycerol. Stamps of these plates were used to grow 100 mL cultures in YPD for DNA extraction. *GAL80* sequencing was done directly on PCR products cleaned by ExoSAP using four primer pairs with at least 100 bp overlap between products, which together covered the sequence of the promoter and *GAL80* ORF. Each PCR amplicon was sequenced in both directions using the PCR primers. Sequences of each colony were assembled, aligned, and analyzed for polymorphisms using the SeqScape V2.6 software (Applied Biosystems) with settings to detect heterozygous loci with a rare allele frequency of 25% or more. Before whole genome sequencing, identified mutations in *GAL80* were sequenced again to verify the identity of the DNA sent for further analysis. In the case that two different *GAL80* mutations were found within one adapted colony, single cells were spread on a plate and 10 representative clones were sequenced to verify the existence of both mutations in different cells.

### Whole genome Illumina sequencing and analysis

Genomic DNA of YPH499N and 39 adapted strains was extracted and subjected to whole-genome resequencing using paired-end sequencing on Illumina Genome Analyzer IIx. Indexing strains for pooling, libraries preparation and sequencing were done as described before [21]. The sequencing reads from all samples were aligned to the reference genome using Novoalign V2.07.18 (http://www.novocraft.com) with parameters -rRandom. The *S. cerevisiae* S288c genome (SGD R64, http://www.yeastgenome.org), along with the sequences of the plasmid, were used as reference genome sequences. The sequencing data for the yeast strains was deposited in NCBI database and can be accessed through BioProject (PRJNA227232, http://www.ncbi.nlm.nih.gov/bioproject/227232) or the Sequence Read Archive (SRA, SRP033016, http://www.ncbi.nlm.nih.gov/sra/?term=SRP033016). SAMtools [22] was used to detect all potential SNPs and Indels for further analysis. Adapted strain specific SNPs were inferred by comparing the frequencies of each nucleotide at each position to the frequencies obtained from the original YPH499N strain. Adapted strain specific Indels were manually checked by comparing to the alignments in YPH499N strain. Whole chromosome duplication was estimated by the read coverage of each chromosome comparing to the whole genome coverage.

## Supporting Information

Figure S1 **Number of adapted colonies per lineage.** A single, naïve, rewired cell was sorted into each well of a 96-well plate containing Glu-his medium. After 48–72 hours incubation, the contents of each well that corresponds to a single lineage were spread on Glu-his agar plates. Colonies were counted after 21 days incubation at 30°C. A histogram of the number of adapted colonies that grew per lineage shows a surprisingly large number of independently adapting sub-lineages (average 19, standard deviation 13.6).
(TIF)

Table S1 **List of 23 mutations found in 28 adapted strains.**
(DOCX)

Movie S1 **Time-lapse microscopy of a single lineage.** Single, naïve, rewired cells plated on Glu-his agar medium were imaged every one hour. Video shows typical dynamics of an adapting lineage grown in this way, depicting continuous progression of adaptation growth of a single lineage. Note that adaptive growth starts at multiple foci within the cell lineage.
(MP4)

## References

[1] Huxley J (1942) Evolution: the modern synthesis. London: Allen & Unwin.

[2] Jablonka E, Raz G (2009) Transgenerational epigenetic inheritance: prevalence, mechanisms and implications for the study of heredity and evolution. The Quarterly Review of Biology 84: 131–176. 19606595 10.1086/598822

[3] Johannes F, Colot V, Jansen RC (2008) OPINION Epigenome dynamics: a quantitative genetics perspective. Nature Reviews Genetics 9: 883–890. 10.1038/nrg2467 18927581

[4] Rando OJ, Verstrepen KJ (2007) Timescales of genetic and epigenetic inheritance. Cell 128: 655–668. 17320504 10.1016/j.cell.2007.01.023

[5] Kondrashov FA, Kondrashov AS (2010) Measurements of spontaneous rates of mutations in the recent past and the near future. Philosophical Transactions of the Royal Society B: Biological Sciences 365: 1169–1176. 10.1098/rstb.2009.0286 PMC2871817 20308091

[6] Lang GI, Murray AW (2008) Estimating the Per-Base-Pair Mutation Rate in the Yeast Saccharomyces cerevisiae. Genetics 178: 67–82. 18202359 10.1534/genetics.107.071506 PMC2206112

[7] Luria SE, Delbruck M (1943) Mutations of bacteria from virus sensitivity to virus resistance. Genetics 28: 491–511. 17247100 10.1093/genetics/28.6.491 PMC1209226

[8] Martincorena I, Luscombe NM (2013) Non-random mutation: The evolution of targeted hypermutation and hypomutation. BioEssays : news and reviews in molecular, cellular and developmental biology 35: 123–130. 10.1002/bies.201200150 23281172

[9] Rosenberg SM, Shee C, Frisch RL, Hastings PJ (2012) Stress-induced mutation via DNA breaks in Escherichia coli: A molecular mechanism with implications for evolution and medicine. Bioessays 34: 885–892. 22911060 10.1002/bies.201200050 PMC3533179

[10] Ram Y, Hadany L (2012) The evolution of stress-induced hypermutation in asexual populations. Evolution 66: 2315–2328. 22759304 10.1111/j.1558-5646.2012.01576.x

[11] Sniegowski PD, Gerrish PJ, Johnson T, Shaver A (2000) The evolution of mutation rates: separating causes from consequences. Bioessays 22: 1057–1066. 11084621 10.1002/1521-1878(200012)22:12<1057::AID-BIES3>3.0.CO;2-W

[12] Sniegowski PD, Murphy HA (2006) Evolvability. Current Biology 16: R831–R834. 17027474 10.1016/j.cub.2006.08.080

[13] Stolovicki E, Dror T, Brenner N, Braun E (2006) Synthetic gene recruitment reveals adaptive reprogramming of gene regulation in yeast. Genetics 173: 75–85. 16510783 10.1534/genetics.106.055442 PMC1461455

[14] David L, Stolovicki E, Haziz E, Braun E (2010) Inherited adaptation of genome-rewired cells in response to a challenging environment. HFSP Journal 4: 131–141. 20811567 10.2976/1.3353782 PMC2929631

[15] David L, Ben-Harosh Y, Stolovicki E, Moore LS, Michelle N, et al (2013) Multiple Genomic changes associated with reorganization of gene regulation and adaptation in yeast. Molecular Biology and Evolution 30: 1514–1526. 23589456 10.1093/molbev/mst071

[16] Stern S, Dror T, Stolovicki E, Brenner N, Braun E (2007) Transcriptional plasticity underlies cellular adaptation to novel challenge. Molecular Systems Biology 3. 10.1038/msb4100147 PMC1865588 17453047

[17] Zeyl C (2004) Capturing the adaptive mutation in yeast. Research in Microbiology 155: 217–223. 15142617 10.1016/j.resmic.2003.12.006

[18] Hall BG (1998) Adaptive mutagenesis: a process that generates almost exclusively beneficial mutations. Genetica 102–3: 109–125. 9720275

[19] Roth JR, Kugelberg E, Reams AB, Kofoid E, Andersson DI (2006) Origin of Mutations Under Selection: The Adaptive Mutation Controversy. Annual Review of Microbiology 60: 477–501. 10.1146/annurev.micro.60.080805.142045 16761951

[20] Stolovicki E, Braun E (2011) Collective Dynamics of Gene Expression in Cell Populations. PLoS ONE 6: e20530. 21698278 10.1371/journal.pone.0020530 PMC3115940

[21] Wilkening S, Tekkedil M, Lin G, Fritsch E, Wei W, et al (2013) Genotyping 1000 yeast strains by next-generation sequencing. BMC Genomics 14: 90. 23394869 10.1186/1471-2164-14-90 PMC3575377

[22] Li H, Handsaker B, Wysoker A, Fennell T, Ruan J, et al (2009) The Sequence Alignment/Map format and SAMtools. Bioinformatics 25: 2078–2079. 19505943 10.1093/bioinformatics/btp352 PMC2723002

