# Supplementary figures and images for: Induced Mutations in Yeast Cell Populations Adapting to an Unforeseen Challenge
Source: PLoS One. 2014 Oct 23;9(10):e111133. doi: 10.1371/journal.pone.0111133 (PMC4207790; doi:10.1371/journal.pone.0111133)

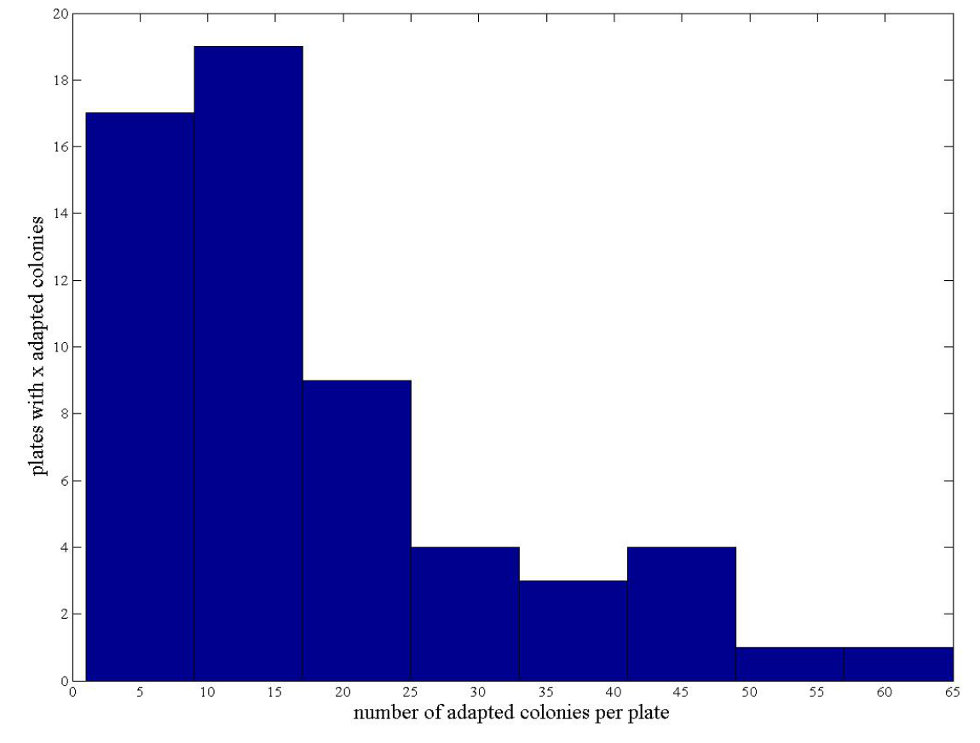

Supplement: Figure S1 — Number of adapted colonies per lineage. A single, naïve, rewired cell was sorted into each well of a 96-well plate containing Glu-his medium. After 48–72 hours incubation, the contents of each well that corresponds to a single lineage were spread on Glu-his agar plates. Colonies were counted after 21 days incubation at 30°C. A histogram of the number of adapted colonies that grew per lineage shows a surprisingly large number of independently adapting sub-lineages (average 19, standard deviation 13.6). (TIF) [file pone.0111133.s001.tif]
