# Supplementary material for: Induced Mutations in Yeast Cell Populations Adapting to an Unforeseen Challenge
Source: PLoS One. 2014 Oct 23;9(10):e111133. doi: 10.1371/journal.pone.0111133 (PMC4207790; doi:10.1371/journal.pone.0111133)
Supplement: Table S1 — List of 23 mutations found in 28 adapted strains. (DOCX) [file pone.0111133.s002.docx]

**Table S1: List of 23 mutations found in 28 adapted strains.**

| Strain | ORF | Gene | Chr. | Position | Ref. DNA | New DNA | Ref. AA | New AA | Type | Gene function |
| --- | --- | --- | --- | --- | --- | --- | --- | --- | --- | --- |
| 100_E4_20 | YBL017C | PEP1 | 2 | 187279 | G | T | D | E | missense | carboxyPEPtidase Y-deficient |
| 100_E4_1 | YDR270W | CCC2 | 4 | 1006148 | C | G | T | T | synonymous | Cross-Complements Ca |
| 200_C10_18 | YDR409W | SIZ1 | 4 | 1291006 | * | TAA |  |  | insertion | SAP and mIZ-finger domain |
| 200_C10_1 | YER036C | ARB1 | 5 | 225128 | A | T | A | A | synonymous | ATP-binding cassette protein involved in Ribosome Biogenesis |
| 100_E4_24 | YER164W | CHD1 | 5 | 505694 | * | A |  |  | deletion | Chromatin organization modifier, Helicase, and DNA-binding domains |
| 100_E4_11 | NA |  | 7 | 67430 | G | A |  |  | intergenic |  |
| 200_C10_2 | YGR285C | ZUO1 | 7 | 1062787 | G | T | Q | K | missense | Ribosome-associated chaperone |
| 200_C10_3 | NA |  | 10 | 123452 | A | G |  |  | intergenic |  |
| 100_E4_11 | YJR140C | HIR3 | 10 | 694525 | A | T | L | * | nonsense | HIstone Regulation |
| 200_C10_11 | YKL209C | STE6 | 11 | 44695 | G | C | S | W | missense | STErile |
| 100_E4_1 | YKL074C | MUD2 | 11 | 295799 | C | T | G | E | missense | Mutant U1 Die |
| 100_E4_21 | NA |  | 11 | 589576 | A | G |  |  | intergenic |  |
| 200_C10_7 | YLR253W |  | 12 | 642737 | G | A | L | L | synonymous |  |
| 100_E4_10 | YML051W | GAL80 | 13 | 172014 | G | T | E | * | nonsense | GALactose metabolism |
| 200_D10_9_6 | YML051W | GAL80 | 13 | 172051 | G | C | R | P | missense | GALactose metabolism |
| 100_F5_8_3 | YML051W | GAL80 | 13 | 172486 | G | A | G | D | missense | GALactose metabolism |
| 100_E4_6 | YML051W | GAL80 | 13 | 172767 | C | T | Q | * | nonsense | GALactose metabolism |
| 100_E4_14 | YMR075W | RCO1 | 13 | 414833 | * | TT |  |  | insertion | Essential subunit of the histone deacetylase Rpd3S complex |
| 100_E4_6 | YNL139C | THO2 | 14 | 361243 | * | GTTGTGAAC |  |  | insertion | suppressor of the Transcriptional defect of Hpr1 by Overexpression |
| 100_E4_24 | YOR009W | TIR4 | 15 | 344934 | * | GCT |  |  | deletion | TIp1-Related |
| 200_D10_9_6 | YOR098C | NUP1 | 15 | 508505 | C | A | A | S | missense | NUclear Pore |
| 200_C10_12 | NA |  | 15 | 769862 | A | C |  |  | intergenic |  |
| 100_E4_24 | YOR301W | RAX1 | 15 | 882245 | C | T | F | F | synonymous |  |
